# Assessment of needle stick and sharp injuries among health care workers in central zone of Tigray, northern Ethiopia

**DOI:** 10.1186/s13104-019-4683-4

**Published:** 2019-10-11

**Authors:** Elias Weldesamuel, Hailay Gebreyesus, Berhe Beyene, Mebrahtu Teweldemedhin, Zemichael Welegebriel, Desalegn Tetemke

**Affiliations:** 1 Department of Public Health, College Health Sciences Hospital, Aksum University, Aksum, Tigray Ethiopia; 2 Department of Medical Laboratory Science, College of Health Sciences & Compreshensive Specialized Hospital, Aksum University, Aksum, Tigray Ethiopia

**Keywords:** Sharp injury, Needle sticks, Health care workers, Health care setting

## Abstract

**Objective:**

Accidental occupational injuries to health care workers (HCWs) continue to have a significant problem in the healthcare system. Thus, the aim of this study was to assess prevalence of needle sticks and sharp injury and associated factors among health care workers working in Central Zone Tigray northern Ethiopia.

**Result:**

The prevalence of needle stick and sharp injury in the past 12 months preceding the study and entire job were 25.9% and 38.5% respectively. Nearly one-third (31%) of the injuries occurred in emergency unit and 122 (71.3%) of the materials caused injury were used on patients. Practice of needle recap, ever used cigarette in last 12 months, training, work hours > 40 per week, job dissatisfaction and work experience less than 5 years were found factors significantly associated with needle stick and sharp injury for health care workers. The magnitude of Needle stick and sharp injury is high in the study area. Policy makers should formulate strategies to improve the working condition for healthcare workers and increase their adherence to universal precautions.

## Introduction

Needle stick and Sharp injuries are a piercing body trauma caused by a sharp medical equipment that was used to screen, diagnose, treat or follow a patient’s disease conditions [1]. Accidental occupational injuries to health care workers (HCWs) continue to have a significant problem in the healthcare system owing to the associated risks [2–5]. Globally, more than 35 million healthcare workers suffer from occupational needle stick and sharp injury every year [6]. Most (86%) occupationally related infections are due to needle stick injury [7, 8]. An estimated 500,000; 100,000 and 600,000–800,000 needle sticks and other percutaneus injuries were reported annually in Germany, UK, and USA HCWs, respectively [9]. The risk of pathogen transmission from an injury with a sharp object has been estimated to be 6–30% for Hepatitis B virus (HBV) in non-immune individuals, 5–10% for Hepatitis C virus (HCV), and 0.3% for human immunodeficiency virus (HIV) [10].

Sub-Saharan African countries have the highest share of HIV prevalence and more than 90% of occupational exposure [11]. Furthermore, the exposure of HCWs to needle stick and sharp injuries (NSSI) causes infections, illness, disability, and death impacting the quality of the healthcare system [12–14]. Studies reported that there is a high prevalence of needle stick injury among health professionals in Ethiopia; Bahrdar 31% [15] and Hawassa 35.8% [16].

The factors associated with NSSI were socio-demographic factors (work experience, educational status, qualification, monthly income, job category), environmental factors (training on infection prevention, long working hours) and other behavioral factors including recapping needles, Use of personal protective equipments (PPE), not complying with standard operating procedures [17–21]. However, previous studies in Ethiopia were limited on referral hospitals and urban settings. Therefore, this study was aimed to assess the prevalence NSSI and its associated factors associated among healthcare workers at health care facilities in central zone, Tigray, northern Ethiopia.

## Main text

### Methods

#### Study area, period, design and population

A facility based cross-sectional study was conducted from February 6, to April 15, 2017 in selected health care facilities of central zone of Tigray which is located approximately 1024 km away from Addis Ababa, Ethiopia. It owns three urban and nine rural districts with a total population of 1,352,557: (663,191 males). In this zone there are 3 general hospitals, 1 comprehensive specialized referral hospital, 6 primary hospitals, 54 health centers and around 200 health posts with 1582 health care workers. All health professionals and auxiliary workers at governmental health care facilities of central zone of Tigray were the source population. From the selected health care facilities, all health care professionals who perform medical procedures on a daily basis, auxiliary workers who worked for at least 1 year and above were included in the study.

#### Sample size and sampling procedure

The sample size was determined using Open-Epi version 2.3.1 software by taking 19.1% prevalence from previous studies on needle stick and other sharp injury [19], considering 5% marginal error, design effect of 2 and with a contingency of 10%, we obtained 456 respondents. In order to have representative respondents from the two urban and three rural districts, multi-stage sampling technique was used; after the proportional allocation to each health care facility, respondents were selected using simple random sampling.

#### Data collection method and quality control

An anonymous, structured, interview questionnaire was developed after reviewing relevant literatures that reviewed key variables as well as earlier studies on NSSI among health care workers [5, 14–16, 18, 20]. Data were collected using pre-tested structured self administered questionnaire by trained Nurse Professionals under the supervision of medical doctors.

#### Data analysis

The pre coded responses were entered in Epi-info version 3.5.3, and then exported to SPSS software version 21 for windows. We have used descriptive statistics to explore the entire variables of the study. Both bi-variable and multi-variable binary logistic regressions were used to identify risk factors for NSSI; variables with *p* value ≤ 0.2 in bi-variable analysis were transferred to a multi-variable analysis to control the effect of confounding. Finally, the 95% CI with p-value of ≤ 0.05 was considered as statistically significant.

### Results

#### Socio-demographic characteristics

From a total of 456 selected health care workers, 444 participated giving a response rate of 97.4%. The mean age of the respondents was 34 (SD ± 9.6) years. The majority of the respondents 213 (48%) were diploma holders in education. The mean time of thier work experience was 11.72 (SD ± 9.8) years (Additional file 1: Table S1). In addition, majority of the respondents were from the outpatient department and nurses in profession (Additional file 2: Fig. S1, Additional file 3: Fig. S2).

#### Participants behavior and working environment

Majority of the respondents 427 (96.2%) were concerned about the health risks of NSSI, of whom the risk was perceived as high in 325 (73.2%) respondents. One hundred sixty-seven (37.6%) respondents had recapped the needle at least once in 12 months preceding the study, and 76 (45.5%) respondents had recapped the needle using their two hands. Nearly all respondents (96.8%) knew the diseases transmitted through needle stick and sharp injuries. The reported prevalence of substance use was 10 (2.3%), 82 (18.5%), 600 (13.5%) and 6 (1.4%) for khat, alcohol, cigarette and other substances respectively (Additional file 4: Table S2).

#### Prevalence and circumstances of needle stick and sharp injury

The overall prevalence of NSSI within the past 2 weeks, 12 months and entire job were 3.8% (95% CI of 2 to 5.6), 25.9% (95% CI of 21.8.0 to 30.2), and 38.5% (95% CI of 34 to 43) respectively. Majority 122 (71.3%) were exposed at the day time (Additional file 5: Table S3). Majority of the injuries were associated with needle stick 115 (67.3%). Nearly one-third (31. %) of the injuries occurred in the emergency unit followed by outpatient (15.2%) and maternity ward (13.5%), Laboratory (9.4%) surgical ward (7%), operation theatre unit (6.4%), medical ward (5.3%), and laundry (5.3%) (Additional file 6: Fig. S3). Majority 122 (71.3%) of the materials that caused the injury were used on patients and 52.4% were from unknown cases (Additional file 5: Table S3). The top reasons for the occurrence of NSSI were sudden movement of the patients during procedure 28.1% followed by disposal and cleaning the work area (18.1%), during giving injection (14.6%), needle recapping 12.3% and drawing blood from a patient 11.7% (Additional file 7: Fig. S4).

#### Factors associated with NSSI within the past 12 months

In the multi-variable logistic regression analysis, the independent factors to needle sticks and sharp injury were, ever smoked cigarette in the last twelve months, training on occupational health and safety practices, working hours per week, job satisfaction and work experience. Study participants who ever practiced needle recap in the last 12 months were 4.3 times more likely to experience injury by needle sticks and other sharps than those who did not recap needles (AOR = 4.326, 95% CI 2.235, 8.373). Health care and auxiliary workers who ever smoked cigarette in the last 12 months were nearly four times more likely to experience needle sticks and sharp injury than those who did not smoke in the last 12 months (AOR = 4.273, 95% CI 1.645, 11.100). Study participants with work experience of less than 5 years were 4.5 times more likely to experience needle sticks and other sharp injury than those with experience of greater than five years (AOR = 4.482, 95% CI 2.189, 9.178) (Table 1).

**Table 1:** Factors associated with occurrence of needle and sharp injury among health care and auxiliary workers at central zone of Tigray, northern Ethiopia, 2017 (n = 444)

Variables/response	NSSI last 12 months		COR (95% CI)	AOR (95% CI)
	Yes n (%)	No n (%)		
Gender				
Male	49 (29.3%)	118 (70.7%)	0.753 (0.489, 1.161)	1.959 (0.959, 4.001)
Female	66 (23.8%)	211 (76.2%)	1	1
Are you concerned about needle sticks and sharp injury				
Yes	114 (26.7%)	313 (73.3%)	1	1
No	1 (5.9%)	16 (94.1%)	5.827 (0.764, 44.443)	7.639 (0.762, 76.602)
Needle sticks and sharp injury is avoidable				
Yes	104 (24.9%)	313 (75.1%)	1	1
No	11 (40.7%)	16 (59.3%)	0.483 (0.217, 1.075)	0.629 (0.188, 2.101)
Disease can be transmitted by needle stick and sharp injury				
Yes	107 (24.9%)	323 (75.1%)	4.025 (1.366, 11.86)^a^	4.479 (0.832, 24.099)
No	8 (57.1%)	6 (42.9%)	1	1
Did you recap needle in the last 12 months				
Yes	73 (43.7%)	94 (56.3%)	4.345 (2.774, 6.806)^a^	4.326 (2.235, 8.373)^b^
No	42 (15.2%)	235 (84.8%)	1	1
Ever used “khat” last 12 months				
Yes	5 (50.0%)	5 (50.0%)	1	1
No	110 (25.3%)	324 (74.7%)	2.945 (.837, 10.367)	4.013 (0.553, 29.142)
Ever used cigarette/tobacco last12 months				
Yes	35 (58.3%)	25 (41.7%)	5.320 (3.011, 9.400)^a^	4.273 (1.645, 11.100)^b^
No	80 (20.8%)	304 (79.2%)	1	1
Did you follow safety guide line properly				
Yes	90 (23.3%)	297 (76.7%)	2.578 (1.452, 4.576)^a^	1.667 (0.733, 3.792)
No	25 (43.9%)	32 (56.1%)	1	1
Ever had training on occupational health and safety				
Yes	102 (46.8%)	116 (53.2%)	1	1
No	13 (5.8%)	213 (94.2%)	14.407 (7.751, 26.78)^a^	14.456 (6.879, 30.375)^b^
Work hours per week				
≤ 40 h	108 (37.5%)	180 (62.5%)	1	1
> 40 h	7 (4.5%)	149 (95.5%)	12.771 (5.769, 28.272)^a^	16.097 (6.252, 41.448)^b^
Job satisfaction				
Satisfied	112 (28.1%)	287 (71.9%)	1	1
Not satisfied	3 (6.7%)	42 (93.3%)	5.463 (1.66, 17.985)^a^	4.264 (1.004, 18.111)^b^
Work experience				
≤ 5 years	25 (14.0%)	153 (86.0%)	3.13 (1.911, 5.125)^a^	4.482 (2.189, 9.178)^b^
> 5 years	90 (33.8%)	176 (66.2%)	1	1

*1* reference, *COR* crude odds ratio, *AOC* adjusted odds ratio, *CI* confidence interval

^a^Significant at COR

^b^Significant at AOR

### Discussion

Healthcare workers are at risk to occupational health hazards mainly due to accidental exposure to injuries such as needle stick and/or other sharp materials [2, 13]. Among health care and auxiliary workers of central Zone of Tigray, the prevalence of NSSI was 25.9% (95% CI of 21.8.0% to 30.2%). This finding is higher than the findings in Bale, Ethiopia 19.1% [18] but in line with the studies done in Bahir-dar 29% and Hawasa 28% [17, 19]. This finding was found to be lower than the findings from Hawasa 35.8%, eastern Ethiopia 31% and Jima 44.12% [14, 22, 23]. The possible difference in the magnitude of the injury could be due to the difference in the capacity of the health care facilities, study participants, and the year of the study. However, whatever the difference in the magnitude of NSSI, studies have implicated that healthcare workers are at higher risk of blood borne pathogens such as HIV, HBV, HCV [2, 24]. This study revealed that needle stick was the major instrument for injury (67.3%) which is in agreement with the previous studies conducted in Bahir-dar (77.3%), and Bale (69.8%).

In this study, six independent factors have been found to be significantly associated with needle sticks and sharp injury.Study participants who ever practiced needle recap in the last 12 months were 4.3 times more likely to experience NSSI than those who did not recap needle (AOR = 4.326, 95% CI 2.235, 8.373). This finding is also similar with the studies conducted in Bale, Hawassa and Addis Ababa, Ethiopia [18, 19, 25]. Health care workers who ever used cigarette in the last 12 months were 4.27 times more likely to experience NSSI than those who did not smoke in the last 12 months (AOR = 4.27, 95% CI 1.64, 11.1). Respondents who were not trained on occupational health safety were 14.5 times more likely to experience NSSI than those who were trained (AOR = 14.46, 95% CI 6.88, 30.37) which is similar with the studies done in Tanzania, Gondar and Addis Ababa [21, 25, 26]. According to a study done in Gondar one of the independent predictor of accidental exposure to HIV was lack of training on infection prevention [21]. Similarly, it was evidenced that injection safety training and infection prevention training reduces the risk of getting sharp injuries by 47.9% and 70% respectively [25].

Respondents who work for greater than 40 h per week were 16 times more likely to experience NSSI than those who were working less than or equal to 40 h per week (AOR = 16, 95% CI 6.252, 41.448). This is similar with the findings from Gondar and Addis Ababa [21, 25]. Study participants who were not satisfied by their job were 4.3 times more likely to experience needle sticks and sharp injury than those satisfied (AOR = 4.264, 95% CI, 1.00, 18.11). This finding is also similar with the study conducted in Gondar and Bahir-Dar [15, 21]. This may be due to stress and emotional upset which could results in poor compliance with health and safety issues for the prevention of occupational exposures [21]. Study participants with a work experience of less than 5 years were 4.5 times more likely to experience NSSI than those with an experience of greater than 5 years (AOR = 4.482, 95% CI 2.189, 9.178). This finding is contradicting with study in Bahr-Dar [17]. The possible difference could be due to the difference in the competency of the junior professionals and availability of supportive capacity building programs.

In conclusion, this study revealed that one-fourth of the respondents had needle stick and sharp injury at least once per year; still the magnitude of NNSI is high in the study area. Practice of needle recap, cigarette smoking, lack of training on occupational health and safety, more working hours per week, job dissatisfaction and less work experience were found to be the major identified risk factors. Therefore, policy makers and health care planners should formulate strategies to improve the working condition for healthcare workers and increase their adherence to universal precautions.

## Limitation

The accuracy of the past experince of the rspondents with regard to the ocurence of the NNSI mighthave been affected by recall bias.

## Supplementary information

**Additional file 1: Table S1.** Socio-demographic characteristics of health care and auxiliary workers at central zone of Tigray, northern Ethiopia, 2017 (n = 444).

**Additional file 2: Figure S1.** A bar graph which shows job category of health care and auxiliary workers in central Zone Tigray, northern Ethiopia, 2017.

**Additional file 3: Figure S2.** A bar graph which shows Current working department of health care and auxiliary workers in central Zone Tigray, northern Ethiopia, 2017.

**Additional file 4: Table S2.** Participants behavior and working environment of health care and auxiliary workers at central zone of Tigray, northern astern Ethiopia, 2017.

**Additional file 5: Table S3.** Needle and Sharp handling method of health care and auxiliary workers at central zone of Tigray, northern Ethiopia, 2017.

**Additional file 6: Figure S3.** A bar graph shows place where needle sticks and sharp injury occurred among health care workers in central Zone Tigray, northern Ethiopia, 2017.

**Additional file 7: Figure S4.** A bar graph which shows the reason for the occurrence of needle sticks and sharp injury among health care and auxiliary workers in central Zone Tigray, northern Ethiopia, 2017.

## Data Availability

The datasets in which conclusion taken is available in the form of Microsoft Excel. It is available on requesting.

## Abbreviations

AKU: Aksum University
BBF: blood and body fluid
HBV: Hepatitis B virus
HCV: Hepatitis C virus
HIV: human immunodeficiency virus
IPPS: Infection Prevention and Patient Safety
NSIs: needle sticks injury
NSSIs: needle stick and sharp injuries
WHO: World Health Organization
