# Supplementary material for: Assessment of needle stick and sharp injuries among health care workers in central zone of Tigray, northern Ethiopia
Source: BMC Res Notes. 2019 Oct 11;12:654. doi: 10.1186/s13104-019-4683-4 (PMC6787964; doi:10.1186/s13104-019-4683-4)
Supplement: Supplementary file 1 — Additional file 1: Table S1. Socio-demographic characteristics of health care and auxiliary workers at central zone of Tigray, northern Ethiopia, 2017 (n = 444). [file 13104_2019_4683_MOESM1_ESM.docx]

| Variable | | Frequency (n=444) | | Percent (%) |
| --- | --- | --- | --- | --- |
| **Age in years** | |  | |  |
| 20-24 | | 87 | | 19.6 |
| 25-29 | | 112 | | 25.2 |
| 30-34 | | 49 | | 11.0 |
| 35-39 | | 40 | | 9.0 |
| >=40 | | 156 | | 35.1 |
| **Gender** | |  | |  |
| Female | | 167 | | 37.6 |
| Male | | 277 | | 62.4 |
| **Religion** | |  | |  |
| Orthodox | | 429 | | 96.6 |
| Muslim | | 13 | | 2.9 |
| Others | | 2 | | 0.5 |
| **Educational level** | |  | |  |
| Primary school | | 20 | | 4.5 |
| Secondary school | | 56 | | 12.6 |
| College diploma | | 213 | | 48.0 |
| Degree and above | | 155 | | 34.9 |
| **Work experience** |  | |  | |
| <= 5 years | 178 | | 40.1 | |
| >5 years | 266 | | 59.9 | |
| **Work place** |  | |  | |
| Urban | 322 | | 72.5 | |
| Rural | 122 | | 27.5 | |
| **Marital status** |  | |  | |
| Unmarried | 122 | | 27.5 | |
| Married | 278 | | 62.6 | |
| Divorced | 32 | | 7.2 | |
| Widowed | 12 | | 2.7 | |
| **Currently working health institution type** |  | |  | |
| General hospital | 159 | | 35.8 | |
| Primary hospital | 86 | | 19.4 | |
| Health center | 199 | | 44.8 | |
| **Monthly income** | | | | |
| <1000 | 84 | | 18.9 | |
| 1000-300 | 220 | | 49.55 | |
| >3000 | 140 | | 31.5 | |
