# Supplementary material for: Assessment of needle stick and sharp injuries among health care workers in central zone of Tigray, northern Ethiopia
Source: BMC Res Notes. 2019 Oct 11;12:654. doi: 10.1186/s13104-019-4683-4 (PMC6787964; doi:10.1186/s13104-019-4683-4)
Supplement: Supplementary file 4 — Additional file 4: Table S2. Participants behavior and working environment of health care and auxiliary workers at central zone of Tigray, northern astern Ethiopia, 2017. [file 13104_2019_4683_MOESM4_ESM.docx]

| Variable/ response | | Frequency (n) | Percent (%) |
| --- | --- | --- | --- |
| Are you concerned about the risk of needle stick/sharp injury (n=444) | Yes | 427 | 96.2 |
|  | No | 17 | 3.8 |
| How do you rate the risk of needle stick/ sharps injuries? (n=444) | Not risky | 15 | 3.4 |
|  | Low risk | 18 | 4.1 |
|  | Moderate risk | 86 | 19.4 |
|  | High risk | 325 | 73.2 |
| Is needle stick and sharps injury avoidable? (n=444) | Yes | 417 | 93.9 |
|  | No | 27 | 6.1 |
| Is disease transmitted by needle & sharp injuries? (n=444) | Yes | 430 | 96.8 |
|  | No | 14 | 3.2 |
| Did you recap needles after use in the last 12 months? (n=444) | Yes | 167 | 37.6 |
|  | No | 277 | 62.4 |
| How often did you recap needles in the last 12 months? (n=167) | Rarely | 41 | 24.6 |
|  | Sometimes | 76 | 45.5 |
|  | Mostly | 18 | 10.8 |
|  | All the time | 32 | 19.2 |
| How did you used to recap the needles after use? (n=167) | With one hand | 91 | 54.5 |
|  | Using two hands | 76 | 45.5 |
| Do you use personal protective equipments? (n=444) | Yes | 346 | 77.9 |
|  | No | 98 | 22.1 |
| Did you wear gloves during the last health care procedure? (n=444) | Yes | 409 | 92.1 |
|  | No | 35 | 7.9 |
| Ever used khat in the last 12 months? (n=444) | Yes | 10 | 2.3 |
|  | No | 434 | 97.7 |
| Ever used alcohol in the last 12 months(n=444) | Yes | 82 | 18.5 |
|  | No | 362 | 81.5 |
| Ever used cigarette/tobacco in the last 12 months (n=444) | Yes | 60 | 13.5 |
|  | No | 384 | 86.5 |
| Ever used substances like hashish, cannabis, heroin, and cocaine in the last 12 months (n=444) | Yes | 6 | 1.4 |
|  | No | 438 | 98.6 |
| Was safety box available at your work place throughout the year? (n=444) | Yes | 402 | 90.5 |
|  | No | 42 | 9.5 |
| Are safety guidelines available at your work environment?(n=444) | Yes | 344 | 77.5 |
|  | No | 100 | 22.5 |
| Did you follow the safety guidelines properly (n=444) | Yes | 387 | 87.2 |
|  | No | 57 | 12.8 |
| Is there a protocol for reporting the injury in your organization? (n=444) | Yes | 242 | 54.5 |
|  | No | 202 | 45.5 |
| Ever had training on occupational health safety?(n=444) | Yes | 218 | 49.1 |
|  | No | 226 | 50.9 |
| Number of hours worked/week (n=444) | Up to 40 hours | 288 | 64.9 |
|  | More than 40 hours | 156 | 35.1 |
| Working time (n=444) | Shift | 83 | 18.7 |
|  | Office hour | 175 | 39.4 |
|  | Both shift and office hour by rotation | 186 | 41.9 |
| Job satisfaction (n=444) | Not satisfied | 45 | 10.1 |
|  | Satisfied | 399 | 89.9 |
