# Supplementary material for: Assessment of needle stick and sharp injuries among health care workers in central zone of Tigray, northern Ethiopia
Source: BMC Res Notes. 2019 Oct 11;12:654. doi: 10.1186/s13104-019-4683-4 (PMC6787964; doi:10.1186/s13104-019-4683-4)
Supplement: Supplementary file 5 — Additional file 5: Table S3. Needle and Sharp handling method of health care and auxiliary workers at central zone of Tigray, northern Ethiopia, 2017. [file 13104_2019_4683_MOESM5_ESM.docx]

| Variables/ Response | | Frequency (n) | Percent (%) |
| --- | --- | --- | --- |
| Have you ever experience needle & sharp injury in your entire job (n=444) | Yes | 171 | 38.5 |
|  | No | 273 | 61.5 |
| Frequency of injury experienced in entire job career (n=171) | 1 time | 78 | 45.6 |
|  | 2 times | 48 | 28.1 |
|  | 3 and above | 45 | 26.3 |
| Have you ever experience needle & Sharps injury in the last 12 months | Yes | 115 | 25.9 |
|  | No | 329 | 74.1 |
| Frequency of sharp injury in last 12 months (n=115) | 1 time | 81 | 69.8 |
|  | 2 times | 24 | 20.7 |
|  | 3 and above | 11 | 9.5 |
| Have you ever experience needle and sharp injury in last 2 weeks(n=444) | Yes | 17 | 3.8 |
|  | No | 427 | 96.2 |
| Frequency of sharp injury in last 2 weeks (n=17) | 1 time | 13 | 76.5 |
|  | 2 times | 4 | 23.5 |
| When do you exposed to the injury (n=171) | Day time | 122 | 71.3 |
|  | Night time | 49 | 28.7 |
| What was the Material caused the injury(n=171) | Needle sticks | 115 | 67.3 |
|  | suturing needle | 14 | 8.2 |
|  | Lancet/Scalpel/blade | 23 | 13.5 |
|  | Glass/other sharp objects | 19 | 11.1 |
| Status of the material cause the injury was (n=171) | used on patient | 122 | 71.3 |
|  | not used on patient | 22 | 12.9 |
|  | not known | 27 | 15.8 |
| If the material caused injury used on patient it was (n=122) | with known case | 59 | 47.6 |
|  | not known case | 65 | 52.4 |
| Did you receive medical care after injury?(n=171) | Yes | 117 | 68.4 |
|  | No | 54 | 31.6 |
| Di you report your injury to concerned body? (n=171) | Yes | 95 | 55.6 |
|  | No | 76 | 44.4 |
| If not reported the reason for not reporting was (n=76) | I don’t think it is important to report | 47 | 61.8 |
|  | no reporting format | 17 | 22.4 |
|  | Fear of stigma | 1 | 1.3 |
|  | patient was low risk for HIV | 8 | 10.5 |
|  | Others | 3 | 3.9 |
