# Supplementary material for: Assessment of needle stick and sharp injuries among health care workers in central zone of Tigray, northern Ethiopia
Source: BMC Res Notes. 2019 Oct 11;12:654. doi: 10.1186/s13104-019-4683-4 (PMC6787964; doi:10.1186/s13104-019-4683-4)
Supplement: Supplementary file 7 — Additional file 7: Figure S4. A bar graph which shows the reason for the occurrence of needle sticks and sharp injury among health care and auxiliary workers in central Zone Tigray, northern Ethiopia, 2017. [file 13104_2019_4683_MOESM7_ESM.docx]

Reasons for the occurrence of NSSI
