# Supplementary material for: Assessment of needle stick and sharp injuries among health care workers in central zone of Tigray, northern Ethiopia
Source: BMC Res Notes. 2019 Oct 11;12:654. doi: 10.1186/s13104-019-4683-4 (PMC6787964; doi:10.1186/s13104-019-4683-4)
Supplement: Supplementary file 5 [file 13104_2019_4683_MOESM2_ESM.docx]

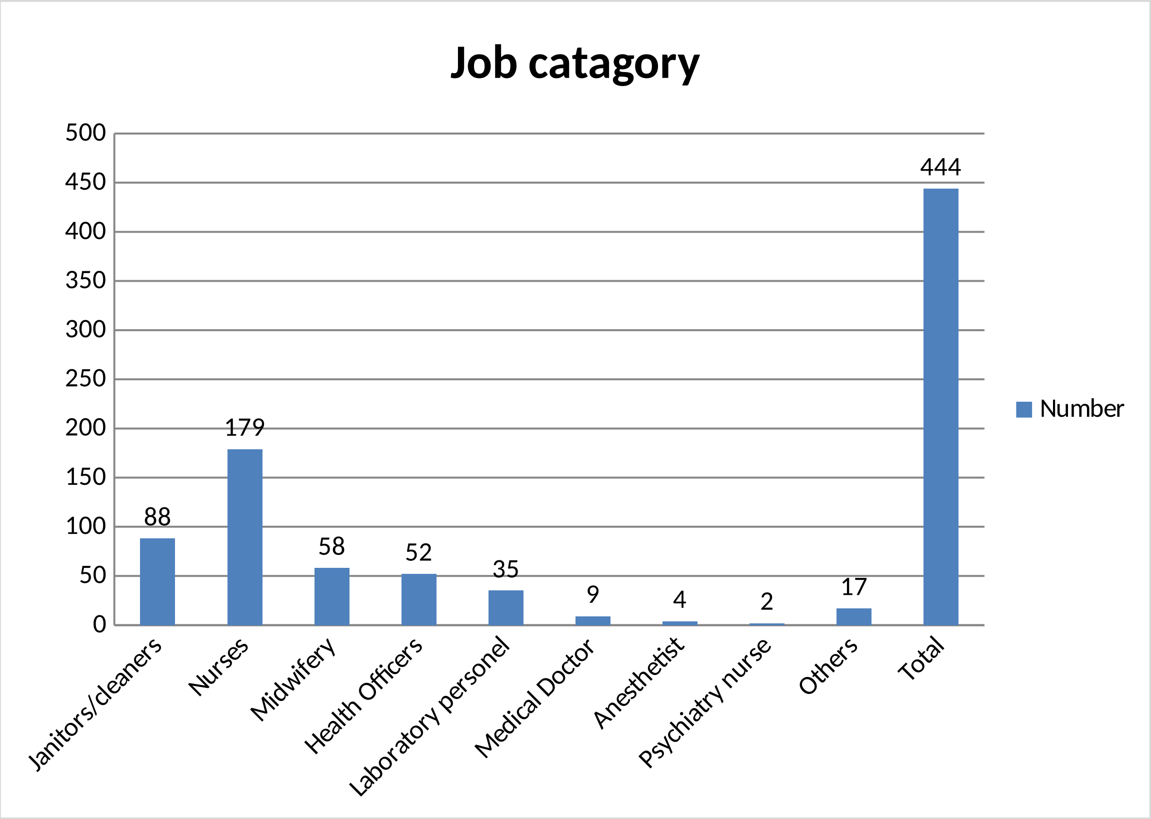
Job catagory
500
450
400
350
300
250
200
150
100
50
0
88
179
58
52
35
9
4
2
17
444
Janitors/cleaners
Nurses
Midwifery
Health Officers
Laboratory personel
Medical Doctor
Anesthetist
Psychiatry nurse
Others
Total
Number
