# Supplementary material for: Assessment of needle stick and sharp injuries among health care workers in central zone of Tigray, northern Ethiopia
Source: BMC Res Notes. 2019 Oct 11;12:654. doi: 10.1186/s13104-019-4683-4 (PMC6787964; doi:10.1186/s13104-019-4683-4)
Supplement: Supplementary file 6 [file 13104_2019_4683_MOESM3_ESM.docx]

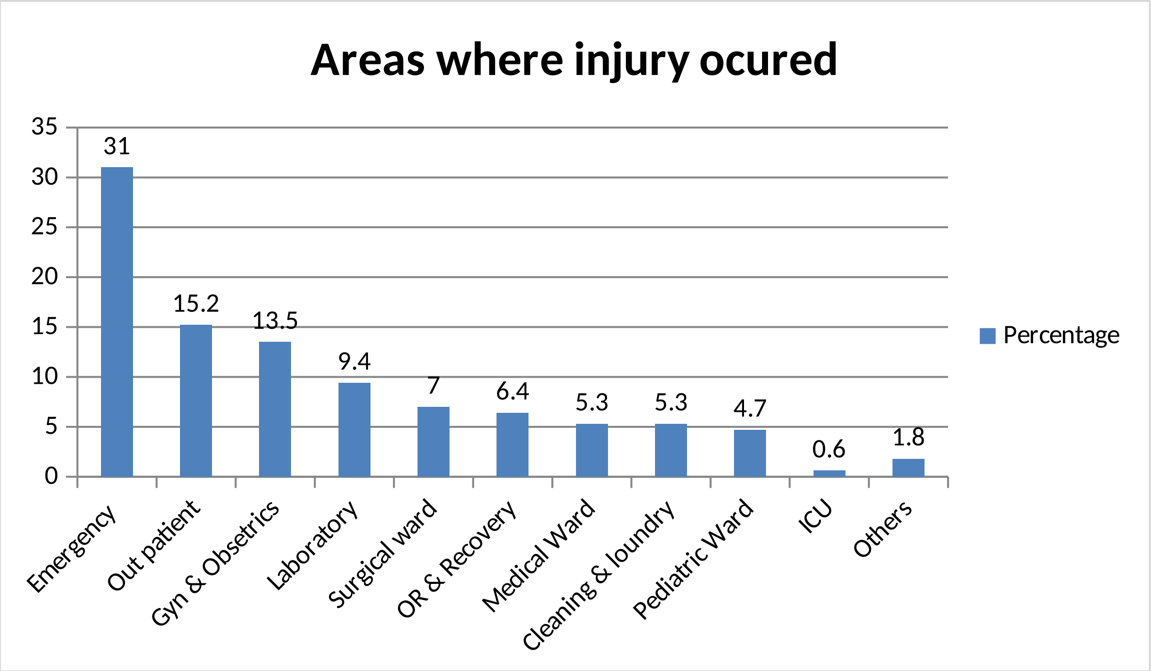
Areas where injury ocured
35
30
25
20
15
10
5
0
31
15.2
13.5
9.4
7
6.4
5.3
5.3
4.7
0.6
1.8
Emergency
Out patient
Gyn & Obsetrics
Laboratory
Surgical ward
OR & Recovery
Medical Ward
Cleaning & loundry
Pediatric Ward
ICU
Others
Percentage
