# Supplementary material for: Assessment of needle stick and sharp injuries among health care workers in central zone of Tigray, northern Ethiopia
Source: BMC Res Notes. 2019 Oct 11;12:654. doi: 10.1186/s13104-019-4683-4 (PMC6787964; doi:10.1186/s13104-019-4683-4)
Supplement: Supplementary file 7 [file 13104_2019_4683_MOESM6_ESM.docx]

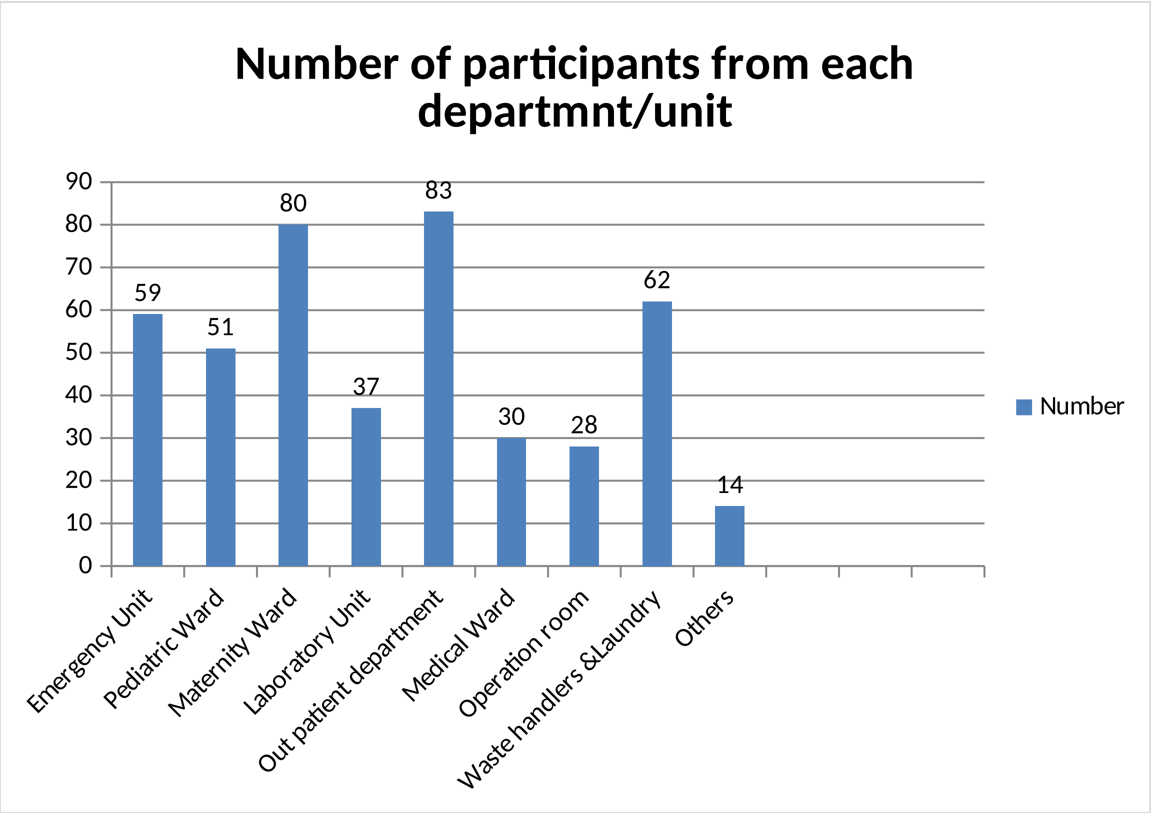
Number of participants from each departmnt/unit
90
80
70
60
50
40
30
20
10
0
59
51
80
37
83
30
28
62
14
Number
Emergency Unit
Pediatric Ward
Maternity Ward
Laboratory Unit
Out patient department
Medical Ward
Operation room
Waste handlers &Laundry
Others
